# A successful response to the second wave of COVID-19 in the slums of Delhi

**DOI:** 10.7189/jogh.15.03032

**Published:** 2025-08-08

**Authors:** John Peteet, Kiran Martin, Jean Peteet

**Affiliations:** 1 Department of Psychiatry, Harvard Medical School, Boston, Massachusetts, USA; 2 Asha Community Health & Development Society, Delhi, India; 3 Department of Health and Rehabilitation Sciences, Boston University, Boston, Massachusetts, USA

## Abstract

The second wave of the COVID-19 pandemic presented unprecedented challenges to India’s slum communities. Although more COVID-related deaths occurred in slum neighbourhoods, the 91 slum communities served by the non-profit Asha Society India experienced only one death case during this period. Key factors contributing to this outcome included the mobilisation of educational, quarantine, and preventive measures, as well as an emergency protocol that was used for early proactive identification and treatment of patients presenting with fever and cough before experiencing shortness of breath indicative of a developing cytokine storm and its life-threatening complications. This retrospective description is limited by the incompleteness of data collected during the chaos of the pandemic.

The second wave of COVID-19 created unexpected difficulties for India and its slum communities, which faced huge shortages of essential hospital aids and supplies – including oxygen – for treating the large numbers of patients. As a result, hospitals turned patients away, while crematoriums ran out of space. Excess mortality in India due to COVID-19 during the period from April to June 2021 was estimated at 2.5–2.8 million, while by 18 May 2021, the country had already reported more than 26.4 million confirmed COVID-19 cases and over 274 000 deaths [1]. Despite high seropositivity, Delhi was among the most-affected cities during the second wave. The rise in new cases was exceptionally rapid in April 2021, increasing from ~2000 to 20 000 between 31 March and 16 April, and was accompanied by a rapid rise in hospitalisations and intensive care unit (ICU) admissions. Even though most of the population had evidence of past SARS-CoV-2 infection, the city of Delhi, with a population of 12.75 million and 4 million slum dwellers living nearby, experienced a massive surge of COVID-19 cases [2], reaching 24 638 new cases a day on 21 April 2021 [2,3].

Research on the COVID-19 response in slum communities has emphasised the difficulty of social distancing among the urban poor [3,4], as well as wide variability in the slum leaders’ ability to gather information about relief schemes, make claims, and attract government responsiveness [5]. However, the Dharavi slum community in Mumbai successfully flattened the curve of the first COVID-19 wave in two months with a proactive screening response strategy and robust surveillance supported by visionary leadership, efficient governance, public-private partnership, and community engagement [2,6].

Similarly, in the first wave of COVID-19, Asha Society India – a community health and development non-governmental organisation established 36 years ago by Dr Kiran Martin, an Indian paediatrician – effectively mobilised 91 Delhi slum communities of 700 000 people by implementing a range of educational, preventive and quarantine measures [7].

During the second COVID-19 wave, as hospitals were overflowing and overwhelmed, the Asha team (consisting of 196 staff and 324 community volunteers, named Asha Corona Warriors) also provided diagnosis and treatment within the slum communities. They faced several challenges: a more infectious and lethal delta variant [8]; vaccine myths (that the vaccine contained pork or caused female infertility), superstitions and shortages; and limited supplies of oxygen, drugs, personal protective equipment, and food (owing to widespread unemployment).

In response to these difficulties, Asha developed an emergency protocol for early diagnosis, quarantining, close monitoring and treatment [9] informed by emerging information about the pathophysiology of the illness [10] and the body’s cytokine response [11,12]. Specifically, on day one, each new patient was assigned a community volunteer to monitor their vital signs and oxygen saturation every two hours and to support the implementation of treatment interventions (*e.g.* sanitising nebulising equipment). If oxygen saturation fell below 95% or if breathlessness accompanied fever, the nurse practitioner or physician prescribed supplemental oxygen, nebulisation, and an individualised dose of oral prednisone (hydrocortisone in two cases), and 5 mg of the anticoagulant apixaban to pre-empt interstitial pneumonia caused by a cytokine storm. Patients receiving apixaban were monitored for potential side effects of easy bruising, nose or gum bleeds, and gastrointestinal bleeding, though none were observed. To prevent indigestion and stomach upset, patients were given one pantoprazole on an empty stomach half an hour before ingesting prednisone. Blood glucose was monitored in patients receiving prednisone, and some patients received insulin for hyperglycaemia. Due to the risk of immunosuppression, blood counts were monitored regularly, and if an elevated white count, purulent sputum, or a rebounding fever after initial improvement indicated the presence of superimposed bacterial pneumonia, patients were prescribed an individualised regimen of azithromycin. However, those who did not respond to the medicine were given augmentin instead. All patients received medicine without waiting for breathlessness to indicate the presence of advancing interstitial pneumonia. Presumptively infected individuals whose oxygen saturation fell below 95% or who had a normal O_2_ saturation and complained of breathlessness were given nebulised corticosteroids and bronchodilators, oral anticoagulants (apixaban), and oxygen using oxygen concentrators donated from the UK. They also received supplementary multivitamins, vitamins D3 (600 000 units) and C, and zinc, which they took for two weeks after recovery. Additional important measures were quarantining to the greatest possible extent, proning, and hydration. The protocol also included masking, meticulous sanitisation of oxygen concentrators, tubing, and nebulisers, monitoring every two hours by a member of the health team, and close attention to co-morbidities. Crucial to its implementation was the availability of oxygen concentrators donated from the UK, along with the organised mobilisation of the Asha team of clinicians (two medical doctors, nine nurse practitioners) and 324 community volunteers known as Asha Corona Warriors.

Community volunteers were trained to record the medical symptoms (*e.g.* cough, weakness) and signs (fever, O_2_ saturation), treatment modalities (oxygen, nebulisers, antibiotics, steroids, supplements), and course of illness on health data cards during six weekly visits (T1–T6) for most individuals presenting with symptoms at Asha’s 20 clinics. During the chaotic early days of the second wave, some individuals who were quarantined were never registered, and most cards lacked complete data. The listed visit times did not indicate when the illness began; in some cases, individuals were examined during the first week of the illness, while in others, visits occurred later.

Demographic and clinical data from 151 of the 1016 cases for which relatively complete and reliable treatment information are summarised in Table S1 in the **Online Supplementary Document**. **Figure 1** shows the frequency of key symptoms, signs, and treatment interventions over time

**Figure 1:**
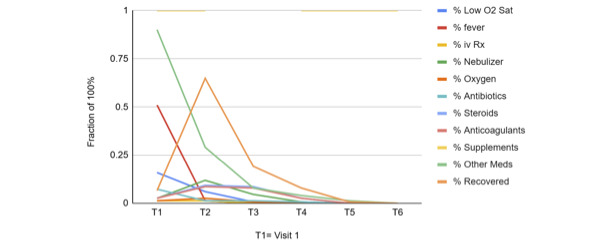
Clinical parameters with time.

Recovery parameters did not appear to differ significantly by age. Furthermore, 85 (95.5%) women and 50 (80.6%) men recovered without worsening. Of the eight individuals with premorbid comorbidities, 1 (12.5%) worsened before recovering, as compared with 5 (3.5%) of individuals without premorbid comorbidities. Two individuals developed comorbidity (elevated blood glucose) during the treatment, both recovering before T5.

Although accurate data are not available, there are more COVID-related deaths in slum neighbourhoods, as would be expected due to higher rates of transmission, greater congestion of health facilities, and less access to private health providers [13]. In contrast, the slum communities served by Asha during the second wave of the COVID-19 pandemic experienced only one death. What could account for this finding?

First, the emergency protocol was developed based on emerging experience with COVID-19 infections. It pivoted from the conventional hospital-based treatment of already seriously ill patients presenting respiratory distress to the proactive identification and early treatment of patients experiencing fever and cough before they experienced shortness of breath indicative of a developing cytokine storm and its life-threatening complications. These data show a pattern of presenting symptoms responding over two to three weeks to intensive interventions begun during week one.

Second, the organisation and extensive deployment of a pre-existing team comprised of health professionals and community volunteers made the effective implementation of preventative measures possible. This included home-to-home screening and surveillance, masking, patrolling of streets, vaccinations, and strategies for dealing with vaccination shortages as described in the 2021 Asha Newsletter [9], as well as an effective emergency protocol.

Third, the strengths of this pre-existing team are grounded in 36 years of work in the community, informed by explicit values of dignity, empowerment, justice, non-violence, compassion, gratitude, generosity, optimism, joy, simplicity, affirmation and touch [13] – which are both lived and professed.

Our report has limitations. Many more individuals were diagnosed and treated than are detailed here due to data being recorded incompletely amid the chaos of the pandemic, possibly introducing selection bias. Although population density did not differ across slums served and information on prior immunity was unavailable, both should be considered as potential confounding factors.

While this model may not be directly translatable to every other slum or low-resource setting, it is worth careful consideration, in preparation for future pandemics.

## Additional material


Online Supplementary Document
