# Supplementary material for: A successful response to the second wave of COVID-19 in the slums of Delhi
Source: J Glob Health. 2025 Aug 8;15:03032. doi: 10.7189/jogh.15.03032 (PMC12333568; doi:10.7189/jogh.15.03032)
Supplement: Online Supplementary Document [file jogh-15-03032-s001.pdf]

Supplement to: Peteet JR, Martin K, Peteet J. A successful response to the second wave of COVID-19 in the slums of Delhi. J Glob Health. 2025;15:03032.

Table S1. Demographic data, co-morbidities, key signs and symptoms, and treatment

[illegible]		[illegible]
Sex		
	Female	89 (58.9)
	Male	62 (41.1)
Age		
	Less than 18	22 (14.6)
	19-49	85 (56.3)
	50+	44 (29.1)
	Total	151 (100)
Co-morbidities		
	Pre-treatment	8 (5.3)
	During Treatment	2 (1.3)
Fever (over 100 degrees)		64 (42.4)
Cough		
	Not a presenting symptom	50 (33.1)
	Presenting symptom	101 (66.9)
	Total with cough	50 (33.1)
Breathlessness		10 (6.6)
Weakness		10 (6.6)
Low O2 Sat (less than 95%)		32 (12.2)
Nebulizer		18 (14)
O2 Treatment		5 (3.3)
Antibiotics		11 (7.3)
Anticoagulants		14 (9.3)
Supplements		7 (4.6)
Other Meds		149 (99.3)
